# Guess Who’s Not Coming to Dinner? Evaluating Online Restaurant Reservations for Disease Surveillance

**DOI:** 10.2196/jmir.2998

**Published:** 2014-01-22

**Authors:** Elaine O Nsoesie, David L Buckeridge, John S Brownstein

**Affiliations:** ^1^Children's Hospital Informatics Program, Boston Children's HospitalBoston, MAUnited States; ^2^Department of Pediatrics, Harvard Medical SchoolBoston, MAUnited States; ^3^Network Dynamics and Simulation Science Laboratory, Virginia Bioinformatics Institute, Virginia TechBlacksburg, MAUnited States; ^4^Department of Epidemiology, Biostatistics and Occupational Health, McGill UniversityMontreal, QCCanada; ^5^Surveillance Lab, McGill Clinical and Health Informatics, McGill UniversityMontreal, QCCanada; ^6^Agence de la santé et des services sociaux de Montréal, Direction de santé publiqueMontreal, QCCanada

**Keywords:** population surveillance, restaurants, epidemics, outbreaks

## Abstract

**Background:**

Alternative data sources are used increasingly to augment traditional public health surveillance systems. Examples include over-the-counter medication sales and school absenteeism.

**Objective:**

We sought to determine if an increase in restaurant table availabilities was associated with an increase in disease incidence, specifically influenza-like illness (ILI).

**Methods:**

Restaurant table availability was monitored using OpenTable, an online restaurant table reservation site. A daily search was performed for restaurants with available tables for 2 at the hour and at half past the hour for 22 distinct times: between 11:00 am-3:30 pm for lunch and between 6:00-11:30 PM for dinner. In the United States, we examined table availability for restaurants in Boston, Atlanta, Baltimore, and Miami. For Mexico, we studied table availabilities in Cancun, Mexico City, Puebla, Monterrey, and Guadalajara. Time series of restaurant use was compared with Google Flu Trends and ILI at the state and national levels for the United States and Mexico using the cross-correlation function.

**Results:**

Differences in restaurant use were observed across sampling times and regions. We also noted similarities in time series trends between data on influenza activity and restaurant use. In some settings, significant correlations greater than 70% were noted between data on restaurant use and ILI trends.

**Conclusions:**

This study introduces and demonstrates the potential value of restaurant use data for event surveillance.

## Introduction

Global adoption of the Internet and mobile phone technologies has proven useful for gathering and disseminating data. Various novel data streams using these technologies have been explored as tools for augmenting traditional public health disease surveillance systems. These novel systems typically aim to improve the detection and monitoring of outbreaks in addition to disseminating information to the public and to public health professionals. Examples include search query volume [1], and digital surveillance systems harnessing news reports and social media, such as HealthMap [2,3] and Global Public Health Intelligence Network (GPHIN) [4]. Other innovative surveillance systems have explored the use of over-the-counter medication sales [5], telephone triage records [6], and school absenteeism [7]. The usefulness of these alternative data sources has been evaluated in several studies, especially for monitoring seasonal and pandemic influenza (eg, Besculides et al [8], Vergu et al [9], Yih et al [10], and Bernardo et al [11]). Another example includes the use of digital surveillance systems to mine early reports of an outbreak of acute respiratory infections, which later evolved into a pandemic in 2009 [12]. Similarly, during the recent 2013 H7N9 influenza outbreak in China, social media sites, such as Twitter and Sina Weibo (a Chinese social network site similar to Twitter), provided near real-time information on disease activity [13].

Similar to school absenteeism, over-the-counter medication sales, and volume of telephone triage service data, utilization of online restaurant reservation sites could also serve as a tool for event surveillance. Studies have noted that the percentage of meals consumed outside the home in the United States has increased [14-16]. Therefore, monitoring changes in restaurant use could possibly serve as a leading indicator of disruption resulting from social unrest, including a public health event. In particular, a decrease in restaurant use could serve as an early indicator of a disease-related event. In this study, we evaluated whether a rise in restaurant table availability was associated with an increase in influenza-like illness (ILI).

## Methods

### Data

All data on restaurant use were obtained from OpenTable [17], an online platform where individuals can make table reservations at restaurants with availabilities at different times of the day. The site caters to restaurants in various cities in several different countries with more than 28,000 restaurants in the database at the time of this writing. The number of registered restaurants is available for each city/region and varies over time as new restaurants join and existing ones either close or cancel their registration.

Each day from September 4, 2012 to April 30, 2013, at set times around lunch and dinner, we conducted a search to determine the number of restaurants with tables available for 2 people. To accommodate differences in regions and eating habits, we defined the lunch period as between 11:00 am-3:30 pm and dinner as between 6-11:30 pm. According to OpenTable policy, customers can cancel reservations up to 30 minutes before the reserved time. Therefore, we searched for restaurants with table availabilities 15 minutes before the times of interest. So, for reservations at 2:00 pm, we would search for available tables every day at 1:45 pm. In addition, we searched for restaurants with table availabilities at the hour and at half past the hour. This resulted in 20 distinct search times each day for each of the 10 study regions in the United States and Mexico. In the United States, table availability was examined for restaurants in Boston (Massachusetts), Atlanta (Georgia), Baltimore (Maryland), and Miami (Florida). For Mexico, we monitored table availabilities in Cancun (Quintana Roo), Mexico City (Distrito Federal), Puebla (Puebla), Monterrey (Nuevo Léon), Guadalajara (Jalisco), and the whole of Mexico. Since data were collected every day at the specified times, our observations formed time series curves of availabilities. Monitoring 10 regions at 20 search times resulted in 200 distinct time series.

### Comparison to Data on Influenza Activity

By using data from the recent 2012-2013 severe influenza season, we tested the hypothesis that an increase in influenza activity was associated with a rise in restaurants with table availabilities. Since the number of restaurants in the system varied over time, we focused on the proportion of restaurants with available tables. For each region, the proportion of restaurants with available tables was defined as the number of restaurants with availabilities at time *t* divided by the total number of restaurants on OpenTable at time *t*. First, we examined the data to better understand trends in table availability during the baseline period. The baseline period was from September to October 2012 because influenza season typically runs from November to April in the northern hemisphere [18]. Observations on restaurant use during the baseline period could suggest best times for surveillance. Next, we calculated the average weekly proportion of restaurants with table availability at each sampling time and compared these data to weekly estimates of ILI. Cross-correlations between time series could be affected by bias because of temporal autocorrelation [19]. Bias in this study could be due to low-frequency patterns resulting from fewer numbers of restaurants open at particular hours of the day. Therefore, we applied prewhitening by fitting an autoregressive integrated moving average (ARIMA) model to availabilities, and then filtering the ILI values using the fitted model. The ARIMA model can be described as follows:

*y_t_*=*c*+*Φ_1_y_t−1_*+…+*Φ_p_y_t−p_−θ_1_z_t−1_*−…−*θ_p_z_t−p_*+*z_t_*

where *c* is a constant, *y*_*t*_ is the observation at time *t*, *y*_*t–p*_ are lagged values of the series, and *z*_*t*_ is a white noise process. Correlations were then examined between the residuals of the availabilities model and the filtered ILI values using the cross-correlation function (CCF). Data representing ILI activity was obtained from state surveillance systems [20-23], Google Flu Trends [24] and the Pan American Health Organization (PAHO) [25]. Google Flu Trends data was available at the city level for cities in the United States and at the province level for Mexico. We calculated correlations between city-level Google Flu Trends and availabilities data for cities in the United States. Due to the unavailability of data at the city level, we estimated correlations between Google Flu Trends state-level data and availabilities for the various cities in Mexico. PAHO’s estimated percent positive for influenza data was only available at the country level for Mexico. Weekly percent ILI (% ILI), resulting from physician visits was also available for all states in the United States. Additionally, for illustrative purposes, local polynomial regression fitting (LOESS) was used in smoothing curves presented in the Results. Smoothing was performed to capture the overall trend of the curves for comparison purposes. See Cleveland [26] for additional information on the LOESS model. Bonferroni adjustment was also applied to account for multiple comparisons as needed. The analysis was performed in R (The R Foundation for Statistical Computing, Wien, Austria).

## Results

### Data Summary: Baseline Period

**Table 1:** Mean proportion of restaurants with available tables at lunch (11:00 am-3:30 pm) and dinner (6:00-11:30 pm) times for cities in the United States and Mexico.

Sampling time	Regions, mean (SD)									
	United States				Mexico					
	Atlanta	Miami	Boston	Baltimore	Mexico City	Cancun	Guadalajara	Monterrey	Puebla	Mexico
11:00 am	0.598 (0.032)	0.668 (0.033)	0.595 (0.021)	0.575 (0.029)						
11:30 am	0.594 (0.032)	0.666 (0.033)	0.595 (0.020)	0.572 (0.028)						
12:00 pm	0.592 (0.035)	0.665 (0.035)	0.592 (0.021)	0.572 (0.028)	0.876 (0.054)	0.638 (0.090)^a^	0.896 (0.065)	0.841 (0.067)	0.982 (0.088)	0.777 (0.050)
12:30 pm	0.590 (0.033)	0.665 (0.035)	0.591 (0.022)^a^	0.573 (0.029)	0.878 (0.055)	0.638 (0.089)^a^	0.9 (0.065)	0.849 (0.068)	0.982 (0.085)	0.78 (0.050)
1:00 pm	0.589 (0.036)	0.664 (0.034)^a^	0.595 (0.022)	0.573 (0.029)	0.876 (0.054)	0.638 (0.089)^a^	0.903 (0.064)	0.855 (0.070)	0.981 (0.088)	0.78 (0.049)
1:30 pm	0.614 (0.034)	0.677 (0.032)	0.628 (0.023)	0.626 (0.029)	0.876 (0.054)	0.641 (0.089)	0.904 (0.066)	0.869 (0.072)	0.984 (0.087)	0.781 (0.048)
2:00 pm	0.604 (0.035)	0.684 (0.034)	0.624 (0.024)	0.622 (0.035)	0.876 (0.054)	0.641 (0.089)	0.896 (0.070)	0.87 (0.067)	0.983 (0.092)	0.781 (0.047)
2:30 pm	0.729 (0.045)	0.758 (0.041)	0.771 (0.034)	0.836 (0.040)	0.867 (0.054)	0.689 (0.092)	0.881 (0.070)	0.888 (0.072)	0.992 (0.092)	0.779 (0.046)
3:00 pm	0.906 (0.049)	0.765 (0.048)	0.887 (0.035)	0.877 (0.042)	0.855 (0.063)	0.693 (0.092)	0.859 (0.074)	0.891 (0.069)	0.99 (0.094)	0.771 (0.046)
3:30 pm	0.899 (0.051)	0.863 (0.041)	0.889 (0.038)	0.894 (0.043)	0.829 (0.075)	0.821 (0.121)	0.836 (0.078)	0.893 (0.071)	0.988 (0.099)	0.763 (0.050)
6:00 pm	0.884 (0.051)	0.876 (0.046)	0.846 (0.042)	0.877 (0.046)	0.833 (0.056)	0.89 (0.130)	0.858 (0.074)	0.912 (0.074)	0.99 (0.100)	0.773 (0.049)
6:30 pm	0.880 (0.051)	0.871 (0.047)	0.847 (0.040)	0.875 (0.044)	0.815 (0.056)	0.891 (0.132)	0.835 (0.075)	0.892 (0.079)	0.97 (0.107)	0.757 (0.048)
7:00 pm	0.884 (0.053)	0.869 (0.048)	0.860 (0.041)	0.879 (0.045)	0.807 (0.058)	0.891 (0.133)	0.829 (0.080)	0.877 (0.079)	0.961 (0.106)	0.749 (0.052)
7:30 pm	0.888 (0.052)	0.871 (0.048)	0.874 (0.043)	0.882 (0.045)	0.782 (0.056)	0.89 (0.131)	0.813 (0.076)	0.847 (0.080)	0.911 (0.097)	0.725 (0.049)
8:00 pm	0.887 (0.054)	0.873 (0.047)	0.874 (0.044)	0.879 (0.045)	0.775 (0.055)	0.891 (0.131)	0.813 (0.076)	0.839 (0.078)	0.911 (0.098)	0.719 (0.049)
8:30 pm	0.882 (0.051)	0.869 (0.047)	0.871 (0.043)	0.872 (0.045)	0.748 (0.056)	0.89 (0.131)	0.799 (0.078)	0.827 (0.084)	0.89 (0.091)	0.699 (0.048)
9:00 pm	0.863 (0.050)	0.864 (0.047)	0.858 (0.043)	0.845 (0.056)	0.74 (0.055)	0.889 (0.13)	0.786 (0.082)	0.799 (0.092)	0.891 (0.088)	0.69 (0.048)
9:30 pm	0.818 (0.066)	0.838 (0.052)	0.835 (0.045)	0.802 (0.076)	0.694 (0.065)	0.884 (0.131)	0.736 (0.095)	0.759 (0.093)	0.881 (0.089)	0.654 (0.050)
10:00 pm	0.682 (0.163)	0.779 (0.089)	0.721 (0.127)	0.656 (0.170)	0.674 (0.077)	0.883 (0.134)	0.688 (0.095)	0.691 (0.096)	0.865 (0.089)	0.634 (0.058)
10:30 pm	0.539 (0.229)^a^	0.677 (0.143)	0.598 (0.217)	0.520 (0.233)^a^	0.599 (0.077)	0.814 (0.148)	0.619 (0.078)	0.635 (0.098)	0.85 (0.097)	0.571 (0.065)
11:00 pm					0.546 (0.08)	0.774 (0.178)	0.563 (0.067)	0.582 (0.071)	0.776 (0.102)	0.525 (0.067)
11:30 pm					0.501 (0.117)^a^	0.717 (0.183)	0.527 (0.068)^a^	0.551 (0.091)^a^	0.705 (0.189)^a^	0.483 (0.097)^a^

^a^Sampling times with lowest proportion of availabilities.

Table availabilities varied by mealtime (lunch and dinner) and by city. Times with lowest availabilities in some cities could represent preferred dining times. Trends observed during preferred dining times should also be most affected by seasonal deviations in dining. Hereafter, we refer to times with lowest availabilities as “most preferred” and times with highest availabilities as “least preferred.” The most- and least-preferred dining times varied by region. Use of *t* tests suggested significant differences (*P*<.001) in availabilities between most- and least-preferred times across all regions. Additionally, mean availabilities at the most- and least-preferred times were also significantly different at lunch and dinner for most regions (*P*<.05), except for lunch in Monterrey (*P*=.23) and Puebla (*P*=.99). Times with the lowest mean proportion of restaurants with available tables at lunch were 2:00 pm (mean 0.608, SD 0.005) for Atlanta, Miami at noon (mean 0.673, SD 0.016), Boston at noon (mean 0.594, SD 0.009), Baltimore at noon (mean 0.589, SD 0.007), Mexico City at 3:30 pm (mean 0.757, SD 0.044), Cancun at 12:30 pm (mean 0.709, SD 0.048), Guadalajara at 3:30 pm (mean 0.818, SD 0.06), Monterrey at noon (mean 0.824, SD 0.055), Puebla at noon (mean 0.963, SD 0.035), and Mexico at 3:30 pm (mean 0.753, SD 0.035). All regions had the lowest proportion of restaurants with available tables for dinner between 10:30-11:30 pm, which could be because of fewer restaurants being open later in the night, especially for US cities. The mean proportion of restaurants with table availability for the entire study period (September 2012-April 2013) is summarized in Table 1.

### Comparison to Data on Influenza Activity

We calculated 300 correlations between data representing ILI activity and restaurant use. Significant correlations (*P*<.05) between the restaurant table availability data for each region and percent positive for influenza from PAHO, percent ILI from states in the United States, and Google Flu Trends are summarized in Table 2. Correlations presented in Table 2 were calculated from prewhitened time series data. We present correlations at weekly lags of 0 and 1 because significant correlations at lag 0 suggest that increase in ILI were associated with immediate increase in availabilities. On the other hand, significant correlations at a 1-week lag indicate that increases in availabilities were followed by a noted increase in ILI. Understanding the correlation at different lags can inform the use of restaurant utilization data for modeling and forecasting future ILI activity. We present results only at lag 1 because the data are at a weekly resolution and studies have suggested that the mean duration of influenza symptoms is presumably less than 7 days [27].

As noted, all regions had the lowest proportion of restaurants with available tables for dinner between 10:30-11:30 pm during the baseline period. As seen in Table 2, these were also the times with the most significant correlations for Miami, Mexico City, and Mexico. Note that most of the dinnertime correlations for Atlanta and Baltimore were observed earlier in the evening compared to Miami. Overall, the highest correlations (>70%) were recorded for Atlanta at 12:30 pm and 3:00 pm at lag 0, and Baltimore at 7 pm at 1-week lag. More significant correlations were also noted between restaurant table availability and Google Flu Trend data compared to PAHO percent positive for influenza and state ILI data (see Table 2). Significant correlations were recorded between PAHO data and restaurant table availabilities for Mexico at 4 set times. As seen in Table 2, a few correlations were also observed between restaurant table availabilities in US cities and state ILI percentages. State ILI trends are not always identical to trends at the city level, which could explain the lack of significant correlations. No significant correlations were noted for Cancun, Guadalajara, Puebla, and Boston.

**Figure 1:**
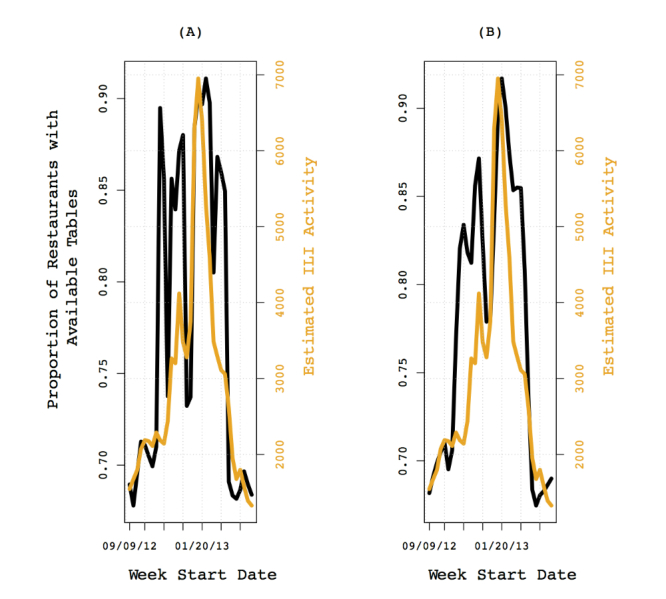
The trend in the proportion of restaurants with table availability at 10:00 PM for Miami compared to Google Flu Trend data for Miami. (A) Restaurant table availability and Google Flu Trend curve; (B) curve with LOESS smoothing to capture the overall trend in availabilities.

**Figure 2:**
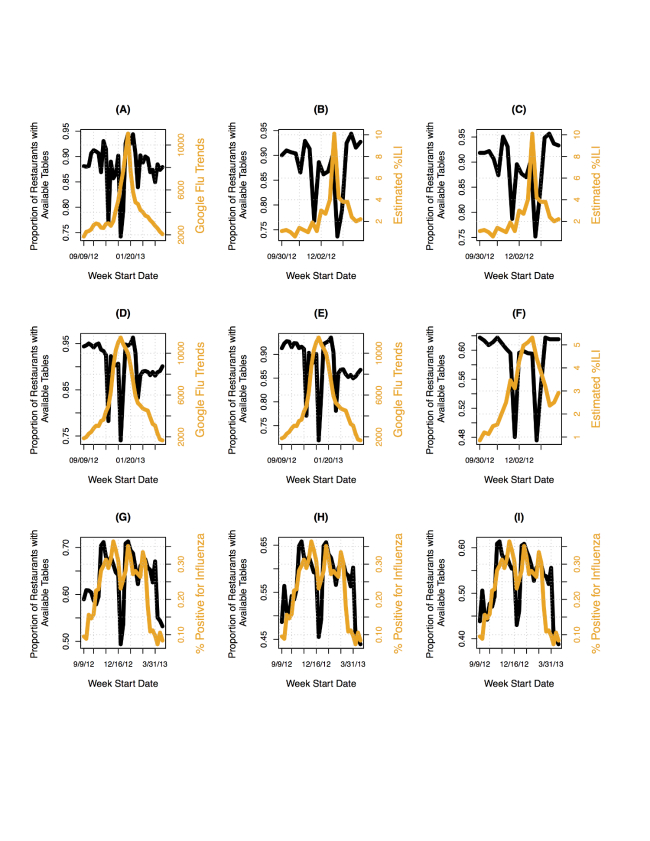
The trend in the proportion of restaurants with table availability compared to trend in various estimates of influenza activity for different regions. (A) Google Flu Trend and restaurant table availability for Baltimore at 7:30 PM; (B, C) estimated % influenza-like illness (ILI) for Maryland and restaurant table availability for Baltimore at 7:00 PM and 3:30 PM, respectively; (D, E) Google Flu Trend and restaurant table availability for Atlanta at 3:00 PM and 6:30 PM, respectively; (F) estimated % ILI for Georgia and restaurant table availability for Atlanta at 12:30 PM; (G-I) Pan American Health Organization (PAHO) % positive for influenza and restaurant table availability for Mexico at 10:00 PM,10:30 PM, and 11:00 PM, respectively.

We present a sample plot showing a case in which the trend in restaurant table availability appears similar to the trend in estimated ILI activity for Miami in Figure 1. Note the dips in the curves before the peak observed during the weeks of Thanksgiving and Christmas holidays. The drop observed after the peak occurred during the week of Valentine’s Day. As seen in Figure 1, the overall trend in the data for availabilities at 10 pm for Miami was similar to that observed with Google Flu Trends, with the peak observed later. This could suggest that an increase in availabilities was observed after a rise in ILI-related queries. Further, 9 graphs representing trends observed during sampling times with the highest correlations for Baltimore, Atlanta, and Mexico are shown in Figure 2. Note the similarities and differences between the curves. In some cases, such as G-I in Figure 2, several of the peaks and troughs in the influenza data are captured by the data on restaurant utilization.

**Table 2:** Significant cross-correlations (*P*<.05) for data source and time for cities in the United States and Mexico.

Country/city		Data source								
		Google Flu Trends			State ILI			PAHO		
		Time	*r* %		Time	*r* %		Time	*r* %	
			Lag 0	Lag1		Lag 0	Lag 1		Lag 0	Lag 1
**United States**										
	Miami	10:30 pm	55.06	58.19	10:30 pm	56.77				
		10:00 pm	53.04							
		9:30 pm		54.56						
		9:00 pm	55.73	65.85						
	Baltimore	7:30 pm		63.60	3:30 pm	63.67				
		8:30 pm		49.93	7:00 pm		70.27			
		8:00 pm		62.75						
	Atlanta	3:30 pm	69.74	46.54	12:30 pm	74.27				
		3:00 pm	70.15							
		6:30 pm	67.25							
		6:00 pm	59.85							
		7:30 pm	55.81	46.60						
		7:00 pm	69.01							
		8:30 pm	66.12	46.40						
		8:00 pm	60.35							
**Mexico**										
	Mexico City	11:30 pm		36.43						
	Monterrey	12:30 pm	51.19	47.43						
	Mexico							10:30 pm		39.83
								10:00 pm		41.57
								11:00 pm		41.76
								11:30 pm		39.33

## Discussion

### Principal Findings

In this paper, we introduced an easily accessible Internet-based data stream—online restaurant reservations—and demonstrated its potential value for event surveillance. More specifically, we observed significant correlations between restaurant table availability and Google Flu Trends, and influenza activity data at the city and country level in the United States and Mexico. In most cases, associations between restaurant use and measures of influenza activity were stronger when all data were at the same geographic resolution. For instance, correlations between restaurant use in Miami and Google Flu Trends data for Miami were stronger than correlations between ILI data for Florida and restaurant use data for Miami. This tendency is explained at least in part by the known variation in ILI trends when measured at different geographical resolutions (eg, city, state, country) [28,29]. We also observed the highest correlations (>70%) for Atlanta at 12:30 pm and 3:00 pm, and for Baltimore at 7 pm. Dinner times with significant correlations were observed later in the evening for regions in the south compared to others. These differences in observations across regions could potentially be explained by differences in dining habits and demographic differences, which could also affect both dining habits and influenza spread [30,31]. Other potential factors include social disruptions, socioeconomic factors, natural disasters, foodborne illnesses, etc. In addition to modeling the data for providing estimates of ILI before the release of official reports, in future studies we would also investigate any occurrences of social unrest and natural disasters, which might have affected the trend in the time series.

### Limitations

Although possibly useful, there are several limitations inherent to the study and the data source impeding a full exploration of this system’s potential. Limitations include differentiating between seasonal changes and changes potentially resulting from a disease-related event. In addition, data on reservation cancellations would be most suitable. However, these data are currently unavailable. This issue could be easily remediated by creating a partnership with restaurants such that occurrences of and reasons for cancellations are recorded through a survey system. Furthermore, only samples of restaurants in each region are listed on OpenTable.

### Conclusions

Despite these limitations, this preliminary analysis suggests that monitoring trends in restaurant table availabilities and cancellations could be useful for detecting social disruption, including disease-related events. Moreover, unlike school absenteeism, over-the-counter medication sales, and volume of telephone triage service data, which are traditionally difficult to access, reservation use data can easily be obtained from reservation sites. The global penetration of the Internet also suggests that such data sources could be easily harvested in the future. These novel data sources could serve as a stepping-stone to prompt further investigation of disease events if warranted. Observations made using this data can be further investigated by comparing trends to other alternative sources for disease surveillance, especially in situations where official reports on disease activity are delayed. Additionally, this data source can be fused with more traditional data streams for epidemic intelligence using ensemble modeling approaches.

## Abbreviations

ARIMA: autoregressive integrated moving average
CCF: cross-correlation function
IARPA: Intelligence Advanced Research Projects Activity
ILI: influenza-like illness
PAHO: Pan American Health Organization
